# *Allopodocotyle palmi* sp. nov. and *Prosorhynchus maternus* Bray & Justine, 2006 (Digenea: Opecoelidae & Bucephalidae) from the Orange-Spotted Grouper *Epinephelus coioides* (Hamilton, 1822) off Bali, Indonesia, Described Using Modern Techniques

**DOI:** 10.1007/s11686-022-00581-x

**Published:** 2022-07-07

**Authors:** Stefan Theisen, Xaver Neitemeier-Duventester, Sonja Kleinertz, Jaydipbhai Suthar, Rodney A. Bray, Patrick Unger

**Affiliations:** 1 Aquaculture and Sea-Ranching, University Rostock, Rostock, Germany; 2 Faculty of Fisheries and Marine Sciences, IPB Bogor Agricultural University, Bogor, Indonesia; 3 Department of Life Sciences, Natural History Museum, London, UK

**Keywords:** ITS2 and 28S rDNA, Phylogeny indicator, Co-evolution, Stenoxenicity, Aquaculture, Hybrid, 3D confocal laser scanning microscopy (CLSM), ‘Palm pattern’

## Abstract

**Background:**

The most convincing species of *Allopodocotyle* Pritchard, 1966 (Digenea: Opecoelidae) are known overwhelmingly from groupers (Serranidae: Epinephelinae). Six species of *Allopodocotyle* have been reported, collectively, from species of *Cromileptes* Swainson, 1839, *Epinephelus* Bloch, 1793 and *Plectropomus* Oken, 1817. These are *A. epinepheli* (Yamaguti, 1942), *A*. *heronensis* Downie & Cribb, 2011, *A*. *manteri* (Saoud & Ramadan, 1984), *A*. *mecopera* (Manter, 1940), *A*. *plectropomi* (Manter, 1963) and *A*. *serrani* (Yamaguti, 1952). In addition, a not yet fully described and unnamed seventh species, morphologically and phylogenetically close to *A*. *epinepheli*, was isolated from the orange-spotted grouper *Epinephelus coioides* (Hamilton, 1822) off Bali, Indonesia in 2016. An eighth species, again from *E*. *coioides* off Bali is described herein.

**Methods:**

Morphological and phylogenetic analyses justify the recognition of *A*. *palmi* sp. nov., which is also genetically different from the as yet unnamed congener from the same host and locality. For the first time, 3D confocal laser scanning microscopy was applied to study and distinguish Digenea taxonomically. We introduce the ‘Palm pattern’, a new simplified way to visualise morphometric differences of related digenean taxa.

**Results:**

*Allopodocotyle palmi* sp. nov. is distinguished from its congeners that infect groupers by its elongate body with a size > 2.7 mm and diagonal testes. The ovary is located mainly, and the anterior testis completely, in the posterior half of the body; the uterine coils are in the fourth eighth of the body. The cirrus-sac is 0.75–1.4 (1.1) mm long, its posterior extremity is well separated from the anterior extent of the vitelline fields, just reaching the anterior border of uterine coils. In addition, *Prosorhynchus maternus* Bray & Justine, 2006 (Bucephalidae) was isolated from *E. coioides*, representing the first record in Indonesia and the third record for this fish species.

**Conclusion:**

The biodiversity research in Indonesia is enhanced with a new species description based on modern and newly applied techniques.

**Supplementary Information:**

The online version contains supplementary material available at 10.1007/s11686-022-00581-x.

## Introduction

The world’s largest archipelago, Indonesia with its > 17,500 islands and a > 95,000 km long coastline covering 5.8 million km^2^, is one of the most populous countries [1, 2]. 60% of the 265 million inhabitants live within 60 km of the coast [2–4]. In 2017, the total area of protected marine and coastal ecosystems has reached 19.1 million ha [3, 5–8]. Since 2010, Indonesia is the top fisheries and aquaculture producer after China [9].

From a current total of 4831 local Indonesian bony fish species including freshwater, 3647 are marine fishes, with more than 2000 being reef-associated [2, 10]. Locally, 47 marine fish families and 138 species are listed to host up to 30 families and 147 species of digenean trematode parasites, not all fully identified [2]. For metazoan parasites, the groupers (Serranidae: Epinephelinae) are among the most intensively surveyed of fishes there. Among the parasitic digeneans, the Opecoelidae and Bucephalidae are two groups for which groupers are important hosts, probably resulting from the attention they received in local parasitological surveys due to their high value [2, 11]. Detailed information on bucephalid trematodes parasitising groupers in Indonesia was provided by Bray et al. [11]. Elsewhere, the trematode fauna of epinephelines is known to be rich, with a handful of families (e.g., the Opecoelidae) and genera (e.g., *Allopodocotyle*) accounting for most of the records [12, 13]. *Epinephelus coioides* is highly valuable commercially. Many individuals from various Indonesian natural habitats and aquaculture facilities have been investigated for parasites (see [2] for detailed summary).

The genus *Allopodocotyle* (Opecoelidae: Hamacreadiinae) contains about 20 species. They are among the most frequently encountered opecoelids in epinephelines in the Indo-West Pacific. Six recognised species infect mainly serranids and are oioxenous or stenoxenous (with uncertain exceptions, see “Discussion” section). These species are *A*. *epinepheli* (Yamaguti, 1942), *A*. *heronensis* Downie & Cribb, 2011, *A*. *manteri* (Saoud et Ramadan, 1984), *A*. *mecopera* (Manter, 1940), *A*. *plectropomi* (Manter, 1963) and *A*. *serrani* (Yamaguti, 1952). They are all parasites of phylogenetically related serranid epinepheline groupers, such as *Cromileptes altivelis* (Valenciennes, 1828) [13], *Epinephelus bruneus* Bloch, 1793 [12, 14], *E*. *chlorostigma* (Valenciennes, 1828) [15], *E*. *cyanopodus* (Richardson, 1846) [16], *E*. *coioides* (Hamilton, 1822) [16–18], *E*. *fasciatus* (Forsskål, 1775) [19], *E*. *fuscoguttatus* (Forsskål, 1775) [13, 17, 18], *E*. *malabaricus* (Bloch & Schneider, 1801) [20], *E*. *quoyanus* (Valenciennes, 1830) [19, 21, 22], *E*. *summana* (Forsskål, 1975) [23], *Plectropomus maculatus* (Bloch, 1790) [24] ‘a large, spotted grouper’ [25], and a *‘Serranus’* sp. [26]. Additionally, a not yet fully described seventh species, morphologically and phylogenetically close to *A*. *epinepheli*, isolated from the orange-spotted grouper *E*. *coioides* off Bali, Indonesia in 2016, remains unnamed (as *Allopodocotyle* sp. B in [16]).

Thirteen species of the Bucephalidae are known from Indonesian waters, including two awaiting full identification [11]. The following species of *Prosorhynchus* Odhner, 1905 have been listed for Indonesia: *P. chorinemi* Yamaguti, 1952, *P. longicollis* Yamaguti, 1953, *P. luzonicus* Velasquez, 1959, *P. platycephali* (Yamaguti, 1934) and *Prosorhynchus* sp. 1 sensu Bray & Palm [27] (syn. *P. australis* sensu Rückert et al. and Palm & Rückert [28, 29]) and *Prosorhynchus* sp. 2 of Bray & Palm [27] (Syn. *P.* cf. *crucibulum* (Rudolphi, 1819) sensu Palm & Rückert [28]).

Whereas standard microscopy and molecular analyses techniques are common in the description of digeneans, 3D confocal laser scanning microscopy as well as the newly introduced and discussed ‘Palm pattern’ have so far not been applied for morphological studies on digenean taxa differentiation [30]. The aim of the present study was to describe a new species of *Allopodocotyle* by implementing new approaches and thereby providing new tools for identifying and distinguishing trematode species. A further aim was to provide a new locality record (*Prosorhynchus maternus*) to add knowledge to the local fish parasite fauna.

## Materials and Methods

### Fish Dissection

One orange-spotted grouper with a total length of 49 cm (standard length 45 cm) and a total weight of 1724 g was obtained from the Kedonganan fish market, South Bali coast, Indonesia on 28th of August, 2019. The fish was transferred on ice to the Marine and Fisheries Faculty Laboratory, Udayana University (UNUD), Kampus Bukit, Jimbaran, Bali, Indonesia. The body cavity was opened and digeneans were collected from the gastro-intestinal system according to the gut wash methodology [31]. Most recovered specimens were stored in 70% EtOH for further morphological analyses (microscopy), others were directly transferred to 99.8% EtOH for molecular analysis.

### Light Microscopy

Digeneans were stained in Mayer-Schuberg’s acetic-carmine solution and mounted in Canada balsam according to a standard protocol [32]. A camera lucida drawing tube was used for illustration and measurements were taken from photographs captured with a digital camera Olympus DP74 attached to an Olympus BX53 DIC light microscope (LM) or a Zeiss SZX10 binocular magnifier, supported with Cellsens 3.2 software (Olympus Soft Imaging Solutions GmbH).

### Confocal Laser Scanning Microscopy

Following a standard protocol [30] selected acetic-carmine stained specimens were visualised with a Leica Stellaris 8 confocal laser scanning microscope at the Institute of Biology, Zoology, Faculty of Mathematics and Natural Sciences, University of Rostock. The following wavelengths were selected to scan the specimens: 514, 568 and 633 nm. The image piles were linked and edited with IMARIS 9.6.7 software (Bitplane, Switzerland) to create three-dimensional images.

### Molecular Analyses and Phylogeny

DNA was extracted and isolated with the Qiagen tissue kit according to the manufacturer instructions. Molecular work was performed according standard protocols [33–35]. For amplifying the second internal transcribed spacer (**ITS2**, applied for the opecoelid species only**)** region, the PCR was run with the primers 3S (5′-GGTACCGGTGGATCACGTGGCTAGTG-3′) and ITS2.2 (5′-CCTGGTTAGTTTCTTTTCCTCCGC-3′) [34, 36]. The protocol for denaturation-annealing-extension cycle was: 3 min at 95 °C, 2 min at 45 °C, 90 s at 72 °C, 4 × (45 s at 95 °C, 45 s at 50 °C, 90 s at 72 °C), 30 × (20 s at 95 °C, 20 s at 52 °C, 90 s at 72 °C) and 5 min extension at 72 °C [34, 36]. Partial **28S** rDNA was amplified using primers ZX-1 (5’-ACCCGCTGAATTTAAGCATAT-3’ and 1500R (5’-GCTATCCTGAGGGAAACTTCG-3’ [37, modified]; using the following cycling conditions: denaturation for 3 min at 94 °C, followed by 40 × (30 s at 94 °C, 30 s at 55 °C, 2 min at 72 °C); and 10 min extension at 72 °C [38, 39]. The **18S** rDNA was amplified using primers WormA (5’-GCGAATGGCTCATTAAATCAG–3’) and WormB (5’-CTTGTTACGACTTTTACTTCC-3’) [40] using the following cycling conditions: denature for 3 min at 94 °C, followed 40 × (30 s at 94 °C, 30 s at 54 °C, 2 min at 72 °C); and 10 min extension at 72 °C [40]. PCR amplicons were purified using the QIAquick PCR Purification Kit (QIAGEN) following the manufacturer’s instructions. Sequences were generated by Seqlab, Germany. Both forward and reverse strands were sequenced, using the amplification primers, but for 18S rDNA, according to a specific protocol [33], PCR products were sequenced using the two PCR primers and internal primers 300F (5’-AGGGTTCGATTCCGGAG-3’ [41]), 600R (5’-ACCGCGGCKGCTGGCACC-3’ [40]), 1270F (5-ACTTAAAGGAATTGACGG-3’ [42]), 1270R (5’- CCGTCAATTCCTTTAAGT-3’ [42]), 1200F (5’-CAGGTCTGTGATGCCC-3’ [4]) and 1200R (5’ GGGCATCACAGACCTG [40]).

Contiguous sequences were assembled and edited in BioEdit Sequence Alignment Editor and Mega X [43, 44]. Representative sequences were submitted to GenBank. For the phylogenetic analyses, the sequences were blasted in NCBI GenBank database and best matching available sequences according to NCBI BLAST were downloaded and aligned. Further, relevant sequences from available phylogenetic analyses were downloaded from the database (see Table 1 and [35]) for information on sequences used). The analyses included all important, comparable sequence data for taxa belonging to the Hamacreadiinae. We did not include data available for species which have previously been implicated with *Allopodocotyle* but do not represent genuine congeners nor members of the Hamacreadiinae. These are *Bathypodocotyle margolisi* (Gibson, 1995) Martin, Huston, Cutmore & Cribb, 2018, previously included in *Allopodocotyle* but now included in the Podocotylinae Dollfus, 1959 (see [35]), represented by 28S (KU320596) data uploaded by Bray et al. [16], and an opecoelid of unknown generic and specific identity from *Scolopsis bilineata* in New Caledonia and Australian waters, represented by ITS2 and 28S data uploaded by Lucas et al. [45] and Bray et al. [16], respectively (see [35]). Outgroup taxa comprised non-Hamacreadiinae opecoelids, specifically *Polypipapiliotrema heniochi* DQ083434 for the ITS2 analysis and *Peracreadium idoneum* AY222209 and *Helicometra epinepheli* KU320597 (originally misidentified as *H. fasciata*) for the 28S analysis. The best fitting phylogeny model was calculated in Mega X, and trees were created following the results of the calculations, for the ITS2 region, the phylogeny was inferred by using the maximum likelihood method based on the Kimura 2-parameter model. For the 28S region the phylogeny was inferred by using the maximum likelihood method based on the General Time Reversible model. The trees with the highest log likelihood are shown. Initial trees for the heuristic search were obtained automatically by applying neighbour-joining and BioNJ algorithms to a matrix of pairwise distances estimated using the maximum composite likelihood (MCL) approach, and then selecting the topology with superior log likelihood value [43, 44]. A discrete Gamma distribution was used to model evolutionary rate differences among sites (5 categories; + G, parameter = 0.3943 for ITS2 and + G, parameter = 0.3226 for 28S). The trees were drawn to scale, with branch lengths measured in the number of substitutions per site [43, 44].

**Table 1:** Species, hosts (snails with family), locality, GenBank accession numbers and references of sequence material for phylogenetic studies (ITS2 and 28S, see Fig. 5)

Species	Host	Locality	References	ITS2 sequence	28S sequence
*Allopodocotyle epinepheli*	*Epinephelus quoyanus*	Great Barrier Reef, Australia	[45]	DQ083423	
*A. epinepheli*	*Salarias fasciatus*	Great Barrier Reef, Australia	[35]	MN067859	
*A. epinepheli*	*Epinephelus cyanopodus*	New Caledonia	[16]		KU320598
*A. heronensis*	*Amblygobius phalaena*	Great Barrier Reef, Australia	[35]	MN067860	
*A. heronensis* (as *A.* sp.)	*Cromileptes altivelis*	Great Barrier Reef, Australia	[35, 45]	DQ083426	
*A. heronensis* (as *A.* sp.)	*Haliotis asinina* (Haliotidae)	Great Barrier Reef, Australia	[35, 45]	DQ083430	
*A. palmi* sp. nov	*Epinephelus coioides*	Bali, Indonesia (fish market)	Present study	OL439064	OL439065
*A.* sp.	*Hemiscyllium ocellatum*	Great Barrier Reef, Australia	[35]	MN067861	
*A.* sp. (same as above, see tree)	*Haliotis asinina* (Haliotidae)	Great Barrier Reef, Australia	[45]	DQ083431	
*A.* sp. B sensu Bray et al. 2016	*Epinephelus coioides*	Bali, Indonesia	[16]		KU320607
*Bentholebouria blatta*	*Pristipomoides argyrogrammicus*	New Caledonia	[16]		KU320606/8
*B. colubrosa*	*Pristipomoides aquilonaris*	West Florida Shelf, USA	[46]		KJ001207
*Cainocreadium dentecis* (as Opecoelidae gen. sp.)	*Haliotis tuberculate* (Haliotidae)	Corsica, Mediterranean	[47]	AJ241806	
*C. dentecis* (as *C. labracis*)	*Dentex dentex*	Corsica, Mediterranean	[47]	AJ241795	
*C. epinepheli*	*Epinephelus cyanopodus*	Great Barrier Reef, Australia	[45]	DQ083428	
*C. epinepheli*	*Epinephelus ongus*	Great Barrier Reef, Australia	[45]	DQ083427	
*C. labracis*	*Gibbula adansonii* (Trochidae)	Rio Ebro Delta, Spain, Mediterranean	[47]	JQ694148	JQ694144
*C. labracis* (as Opecoelidae gen. sp.)	*Jujubinus striatus* (Trochidae)	Corsica, Mediterranean	[47]	AJ241808	
*C. lintoni*	*Epinephelus morio*	Caribbean Sea, off Virgin Islands	[46]		KJ001208
*C.* sp. (as Opecoelidae gen. sp.)	*Steromphala adansonii* (Trochidae)	Corsica, Mediterranean	[47]	AJ241807	
*Hamacreadium cribbi*	*Lethrinus miniatus*	New Caledonia	[16, 35]		KU320603
*H. mutabile*	*Lutjanus griseus*	West Florida Shelf	[46]		KJ001209
*H. mutabile*	*Lutjanus fulviflamma*	New Caledonia	[16]		KU320601
*H.* sp. 1 SM-2019	*Lutjanus carponotatus*	Great Barrier Reef, Australia	[35]		MN067857
*H.* sp. 1 SM-2019	*Lutjanus gibbus*	Great Barrier Reef, Australia	[35]		MN067856
*H.* sp. 2 SM-2019	*Lutjanus argentimaculatus*	Great Barrier Reef, Australia	[35]		MN067858
*H.* sp. A	*Salarias fasciatus*	Great Barrier Reef, Australia	[35]	MN067862	
*H.* sp. B	*Neoglyphidodon melas*	Great Barrier Reef, Australia	[35]	MN067864	
*Helicometra epinepheli* (as *H. fasciata*) (outgroup)	*Epinephelus fasciatus*	New Caledonia	[35]		KU320597 (outgroup)
*Macvicaria macassarensis*	*Lethrinus miniatus*	Australia	[38]		AY222208
*Pacificreadium serrani*	*Plectropomus leopardus*	Great Barrier Reef, Australia	[45]	DQ083433	
*P. serrani*	*Plectropomus leopardus*	New Caledonia	[16]		KU320602
*P. serrani*	*Thalassoma lunare*	Great Barrier Reef, Australia	[16]	MN067865	
*Peracreadium idoneum*	*Anarhichas lupus*	United Kingdom	[38]		AY222209
*Podocotyle scorpaenae*	*Scorpaena scrofa*	Corsica, Mediterranean	[47]	AJ241794	
*P. scorpaenae* (as Opecoelidae gen. sp.)	*Clanculus jussieui*	Corsica, Mediterranean	[47]	AJ241809	
*Podocotyloides australis*	*Diagramma labiosum*	Great Barrier Reef, Australia	[48]	MF805694	
*P. brevivesiculatus*	*Plectorhinchus lineatus*	Great Barrier Reef, Australia	[48]	MF805697	
*P. gracilis* (as *Allopodocotyle* sp.)	*Diagramma labiosum*	Great Barrier Reef, Australia	[45]	DQ083422	
*P. gracilis*	*Diagramma labiosum*	Great Barrier Reef, Australia	[48]	MF805691	
*Polypipapiliotrema heniochi* (outgroup)	*Heniochus chrysostomus*	Great Barrier Reef, Australia	[45]	DQ083434 (outgroup)	

### Palm Pattern

The line drawing of the *Allopodocotyle* specimen as well as figures of congeners from their original descriptions were scanned and opened in Adobe Photoshop CS5 separately. When specimens were not mounted and drawn straight, but with a partly bent body (e.g., forebody), such parts were cropped and rotated until individuals were positioned straight without changes in the body character lengths and their ratios. Total body length was visualised by a black bar, and coloured smaller bars were inserted to show the longitudinal position and range of the organs. We term this diagrammatic representation of a digenean a ‘Palm pattern’. When comparing digenean species, it is useful to consider both actual and proportional size and position of features. Thus, we present both standardised and to-scale Palm patterns. All Palm patterns of the species were compared by adjusting the relative length of the body. Therefore, the real interspecific size is not comparable in these so called absolute Palm patterns, but the position of the organs is. The bodies (black bars) were longitudinally divided into eighths for easier comparison of organ positions. Finally, all absolute Palm patterns were compared for their actual proportions. The congeners were orientated with the middle of their bodies on a longitudinal axis. This comparison with respect to the actual body length of the taxa shows the herewith defined relative Palm patterns.

### Nomenclatural Acts

The description of *Allopodocotyle palmi* sp. nov. (life science species identifier number (ZooBank LSID): LSID urn:lsid:zoobank.org:act:96F57820-2E6E-432A-8468-715F033778FC) complies with the requirements of the International Commission on Zoological Nomenclature (ICZN). The LSID for this publication is: LSID urn:lsid:zoobank.org:pub:0A1D052A-DF99-41EE-B2AA-EA6F9BDBF5F7. The electronic edition of this work was published in a journal with an ISSN and has been archived and is available from digital repositories. DNA sequences are available in GenBank under the GenBank accession numbers OL439065 (*Allopodocotyle* 28S) and OL439064 (*Allopodocotyle* ITS2) and OL439126 (*Prosorhynchus*).

## Results

### Taxonomy and Description

Family **Opecoelidae Ozaki, 1925.**

Genus ***Allopodocotyle* Pritchard, 1966**

Synonym: *Pedunculotrema* Fischthal & Thomas, 1970

Type species: *Allopodocotyle plectropomi* (Manter, 1963) Pritchard, 1966

***Allopodocotyle palmi*** **sp. nov.**

LSID urn:lsid:zoobank.org:act:96F57820-2E6E-432A-8468-715F033778FC

**Fig. 1:**
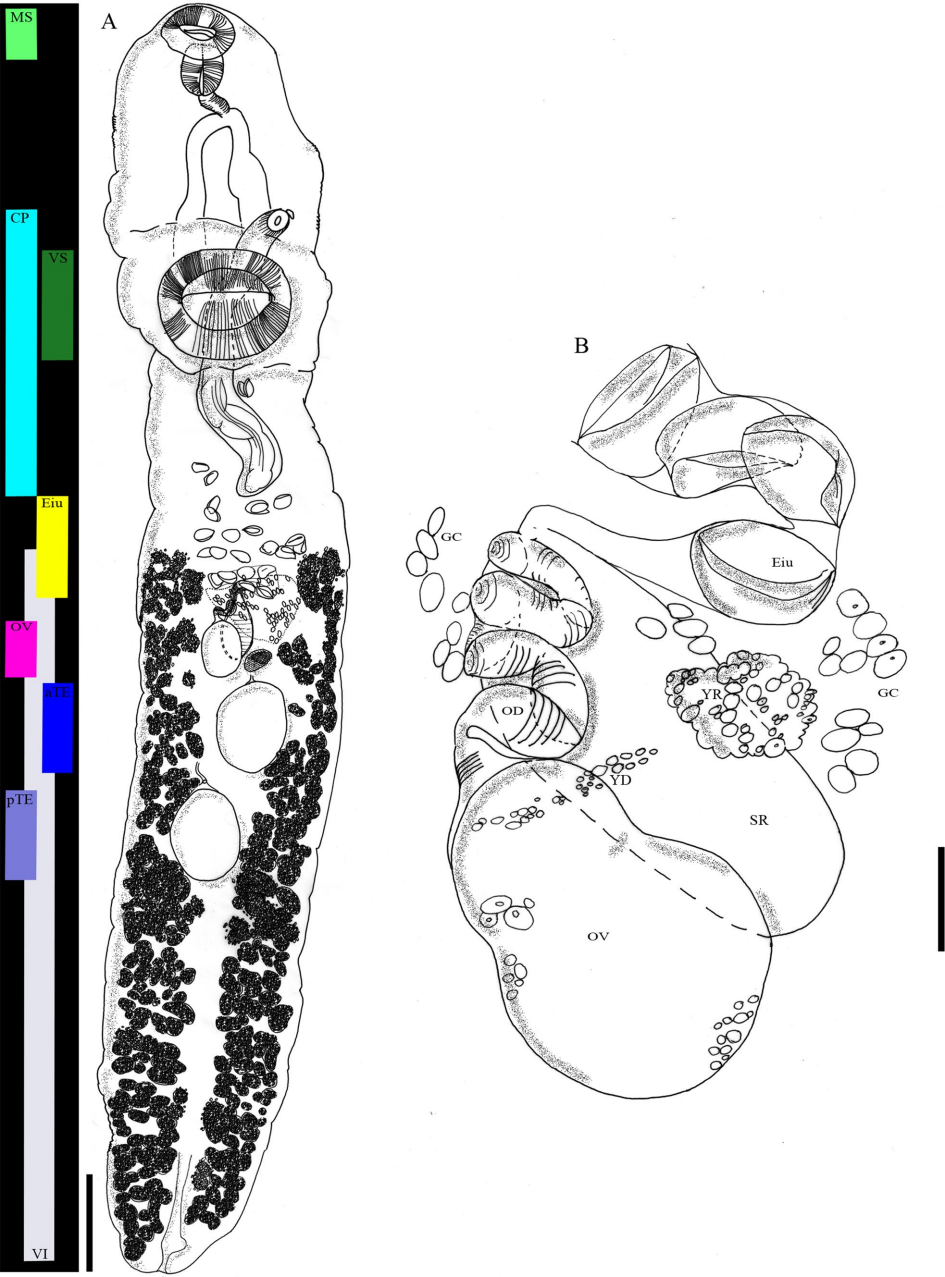
*Allopodocotyle palmi* sp. nov. line drawings and Palm pattern. **A** Habitus, dorsoventrally flattened and longitudinal Palm pattern **B** Detail of the female reproductive system with ovary (OV), seminal receptacle (SR), oviduct (OD), yolk reservoir (YR) and yolk duct (YD), and eggs in utero (Eiu), surrounded by (Mehlis’) gland cells (GC); scale bars: 300 µ in (**A**), 50 µ in (**B**)

**Fig. 2:**
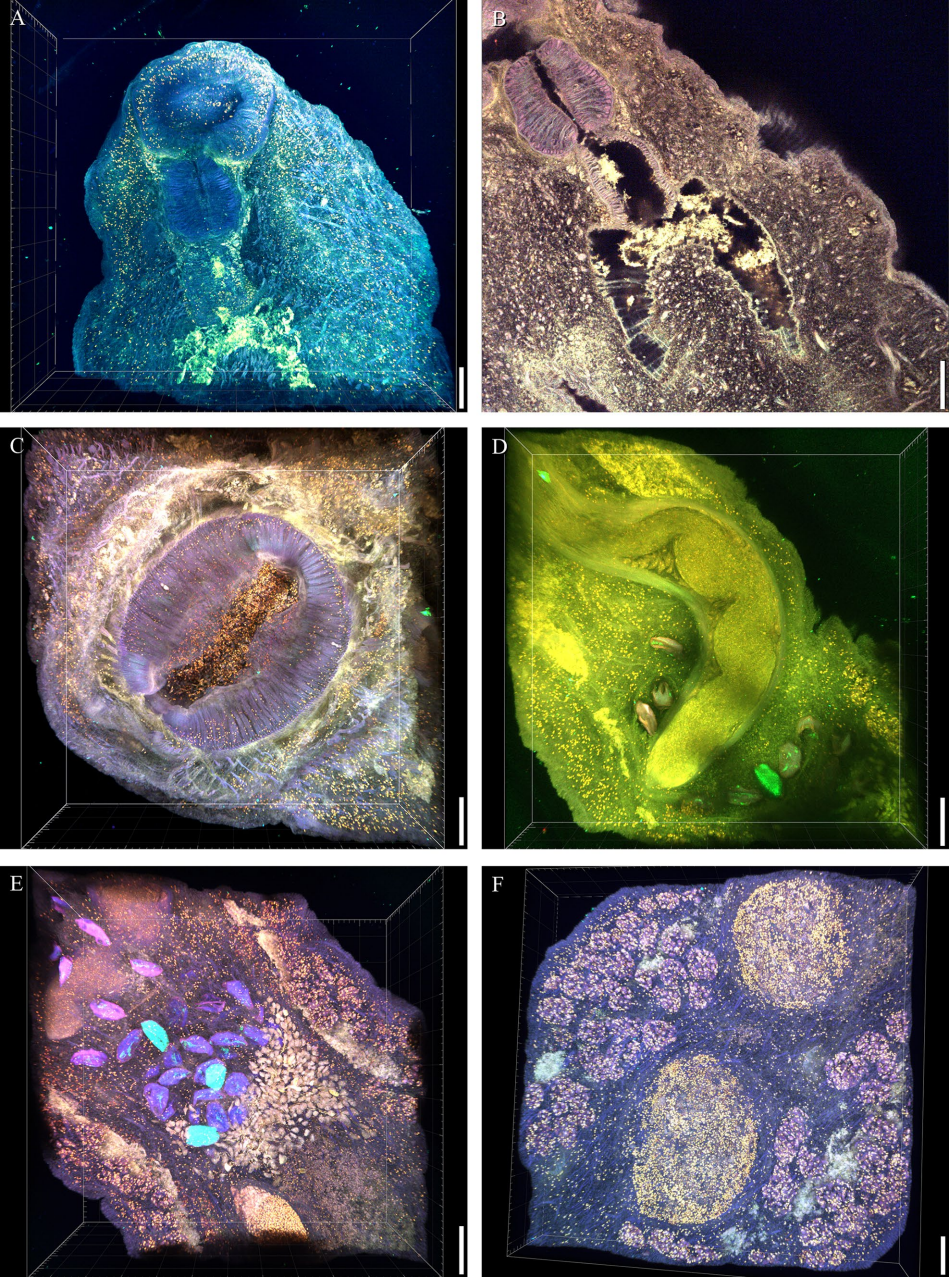
*Allopodocotyle palmi* sp. nov. Confocal Laser Scanning Microscopy. **A** Anterior with mouth sucker followed by pharynx (dark blue), oesophagus and intestinal bifurcation (bright green). **B** Detail of lumen in muscular pharynx, oesophagus and intestinal bifurcation. **C** Ventral sucker. **D** Cirrus-sac with ejaculatory duct in anterior narrow portion. **E** Eggs and (Mehlis’) gland cells anterior to ovary. **F** Vitelline follicle fields and testes; scale bars 60 µ in (**A**, **B** and **F**), 80 µ in (**C**, **D** and **E**)

**Fig. 3:**
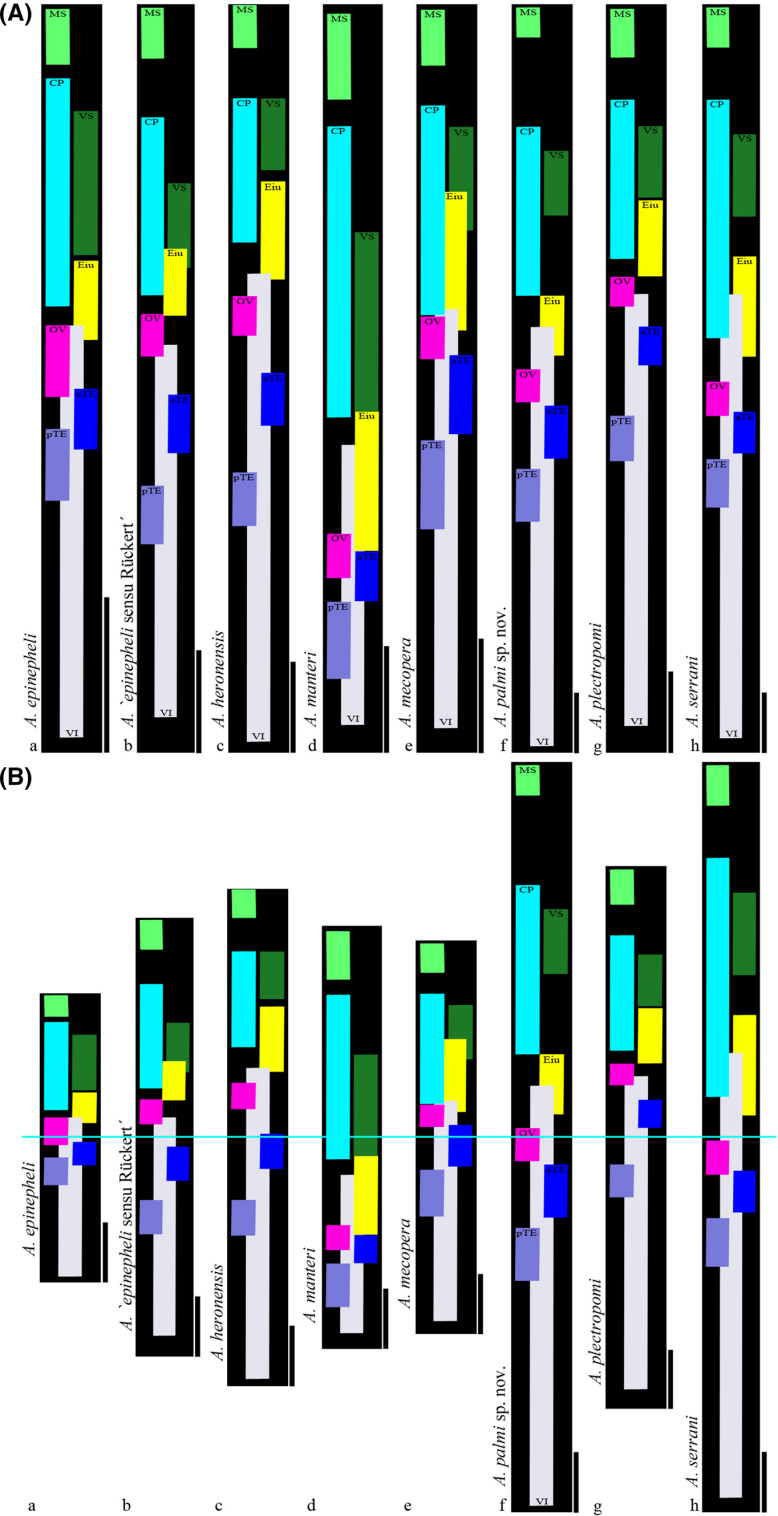
‘Palm pattern’ of grouper-infecting *Allopodocotyle* spp. **A** Absolute Palm patterns of congeners. **B** Relative Palm patterns set in size relation (from original drawings e.g., of holotypes) to each other for direct interspecific comparison. Scale bars 300 µ (originally published sizes of *A*. *manteri* are not 100% equal to the scaled associated original line drawing that we refer to)

***Type-host:*** *Epinephelus coioides* (Hamilton, 1822), orange-spotted grouper, local name: Kerapu (grouper) lumpur (mud)
***Type-locality:*** Kedonganan fish market, South Bali coast, Indonesia
***Habitat:*** Intestine, pyloric caeca
***Type-material:*** Holotype MZBTr 257; paratypes MZBTr 258–261 and additional paratypes E.7653–E.7658
***Deposition of specimens:*** Zoological Museum Bogor, Indonesia, numbers MZBTr 257 (holotype) and MZBTr 258–261 (paratypes); Berlin Natural History Museum, Germany (‘Museum für Naturkunde’, catalogue ‘Entozoa’, collection ‘Vermes’), numbers E.7653–E.7658 (additional paratypes)
***Infection:*** One hundred specimens (of them 60 from the intestine and 40 from the pyloric caeca) were isolated from the fish
***Etymology:*** The specific name is to honour Prof. Dr Harry Palm, (former) supervisor of all authors (except Dr Bray) and his work on Indonesian marine fish parasites, especially of groupers
***Description:*** (all μm) (Figs. 1, 2, 3, Table 2 and additional file 1: Table S1): Measurements of 22 gravid whole-mount worms. Body elongate, sides almost parallel, sometimes curved dorsoventrally with curvature of body and protuberance of ventral sucker typically causing specimens to mount laterally, with maximum width in hindbody, typically in region of gonads, 2912–4563 (3669) × 296–714 (477); length to width ratio 5.1–12.1 (7.9):1, tegument unarmed. Forebody 429–1005 (717) long, occupies 11.7–27 (19.7)% of body length. Oral sucker opens ventro-subterminally, 117–188 (149) × 103–222 (160). Ventral sucker subglobular, protuberant, at border of first to second quarter of body, 224–380 (304) × 250–412 (312). Ventral to oral sucker width ratio 1.5–2.8 (2):1, length ratio 1.4–2.9 (2.1):1. Prepharynx not observed (appears to be visible in Fig. 2B). Pharynx subglobular, anterior extremity protrudes slightly dorsal to oral sucker, 94–137 (111) × 75–126 (99). Oesophagus muscular, 82–217 (130) long, occupies 2.6–5.4 (3.6)% of body length. Intestinal bifurcation in mid-forebody (halfway between anterior extremity and genital pore). Caeca long, blind, terminate close to posterior extremity

**Table 2:** Comparative morphometries of grouper-infecting *Allopodocotyle* spp.

Species and measures	*Allopodocotyle palmi* sp. Nov.			*A. epinepheli*	*A. epinepheli* sensu Rückert 2006	*A. heronensis*	*A. manteri*	*A. mecopera*	*A. plectropomi*	*A. serrani*
Source	Present study			[15, 21]	[17, 18]	[13]	[23]	[25]	[24, 49]	[26, 49]
Host	*Epinephelus coioides*			*E. quoyanus*	*E. coioides, E. fuscoguttatus*	*Cromileptes altivelis, E. fuscoguttatus*	*E. summana*	‘Spotted grouper’	*Plectropomus maculatus*	‘*Serranus* sp.’
Locality (type)	Bali, Indonesia			Heron Island, Australia	Bali, Indonesia	Heron Island, Australia	Red Sea, Egypt	James Island, Galapagos	Suva, Fiji	Makassar, Indonesia
*n*	22			2	1 drawing, all data based on drawing	12	5	9	26	2
	Min	Max	Mean							
Length	2912.0	4563.0	3669.4	1340–2050	2300	1903–3287 (2643)	1130–1750	1849–2538	2717–3534	3700–4300
Width	296.0	714.0	477.4	335–369, to 550 in original reference	400	222–380 (310)	350–650	517–600	482–616	800–900
Length to width ratio (:1)	5.1	12.1	7.9		5.8	7.2–11 (8.8)	2.2–3.7	3.6–4.2	4.5 (see second reference)	4.3 (see second reference)
Forebody length	429.0	1005.0	717.3		700	338–705 (568)	360–610	262–375	435–536	
Forebody %	11.7	27.0	19.7	23–24	30.4	17–25 (22)		10–16.7	‘About one-sixth’	18 (see second reference)
Oral sucker length	117.0	188.0	149.1	120–123	150	78–151 (123)	130–180		167–207 diameter	180–190
Oral sucker width	103.0	222.0	160.0	122–133	140	99–149 (128)	140–210	157–188		210–240
Ventral sucker length	224.0	380.0	304.1	199–258, 280–350 in original reference	230	164–301 (233)	310–340		274–301 diameter	400–450
Ventral sucker width	250.0	412.0	312.0	243–297, 310–380 in original reference	260	177–296 (256)	340–450	375–420		470–500
Ventral to oral sucker length ratio (:1)	2.1	1.4	2.9							
Ventral to oral sucker width ratio (:1)	1.5	2.8	2.0	2.0–2.2	1.9	1.2–2.2 (1.9)	Oral to ventral is 0.41–0.54	‘Slightly more than twice’	1.5–1.7	2.2
Pharynx length	94.0	137.0	110.6	97–108, 100–120 in original reference	100	59–114 (92)	40–80 diameter	102–144	134–144	120–140
Pharynx width	75.0	126.0	98.8	82–85, 80–85 in original reference	90	72–106 (93)		70–83	93–113	130–160
Oral sucker to pharynx width ratio (:1)	1.0	2.2	1.6		1.6			2.2–2.3	2.1 (see second reference)	2.9 (see second reference)
Oesophagus length	82.0	217.0	130.4	80–124	100	39–118 (73)		‘Shorter than pharynx’	88–120	100–180
Oesophagus %	2.6	5.4	3.6		4.4	2–4.4 (3)				
Distance between testes	73.5	248.0	140.5	0	120	108–310 (204)	‘Very near’	30	250	85
Anterior testis length	119.0	255.0	179.3	148–189	160	100–246 (181)	80–170	Round		200–340
Anterior testis width	96.0	207.0	151.0	97–123	140	85–216 (160)	80–160			220–300
Posterior testis length	137.0	275.0	197.0	148–166	160	98–246 (183)	90–180			200–340
Posterior testis width	110.0	198.0	160.1	104–123	140	81–226 (162)	100–170			220–300
Post-testicular region	876.0	1739.0	1209.8	328–558	650	617–1151 (822)		600–900	798–1330	
Post-testicular region %	28.9	39.2	32.9		28.7				‘More than one-third body length’	
Cirrus-sac length	751.0	1438.0	1045.8	335, 450–480 in original reference	600	510 based on drawing	210–310		903 (see second reference)	1500–1860
Cirrus-sac width	80.0	135.0	96.6	57, 54–100 in original reference	80		50–60			130–140
Ovary length	81.0	179.0	122.5	136–189, 150–200 in original reference	140	76–187 (134)	70–170			220–250
Ovary width	60.0	132.0	91.6	79–88, 100–145 in original reference	90	71–144 (101)	90–120			120–200
Pre-vitelline region	1173.0	1969.0	1565.3		1050	389–1089 (811)				
Pre-vitelline region %	38.3	46.9	42.7		45.6					
Range of follicles	1652.0	2697.0	2121.1		1150	Posterior extremity to anterior to ovary				
Cirrus-sac reach	2272.0	3919.0	3046.3							
Cirrus-sac reach %	78.0	86.2	82.9							
Eggs length	53.0	72.0	62.2	68–70	65	48–68 (59)	65–77	70–83	64–68	63–66
Eggs width	30.0	56.0	42.7	32–38	35	30–44 (37)	46–52	32–48	32–40	36–39

Testes two, diagonal, oval, entire, in centre of hindbody, separated by 73.5–248 (141). Anterior testis 119–255 (179) × 96–207 (151). Posterior testis 137–275 (197) × 110–198 (160). Post-testicular region 876–1739 (1210), occupies 28.9–39.2 (32.9)% of body length. Cirrus-sac extends from midway between ovary and posterior margin of ventral sucker to genital pore, distinctly swollen posterior to ventral sucker, narrower dorsal to ventral sucker, containing winding internal seminal vesicle posteriorly, 751–1438 (1046, occupies 28.5% of mean body length) × 80–135 (97). Cirrus-sac reach, defined as distance between posterior extremity of worm and anterior most extent of cirrus-sac (here = genital pore), 2272–31,919 (3046), counting for 78–86.3 (82.9)% of body length. Distinct pars prostatica not observed. Ejaculatory duct a straight, narrow tube apparently extending length of anterior narrow portion of cirrus-sac. Genital atrium small but distinct. Genital pore sinistral, directly anterior to ventral sucker at base of its protuberance. Ovary 81–179 (123) × 60–132 (92). Saccular seminal receptacle anterodorsal to ovary. Yolk reservoir sinistral to ovary anterior to anterior testis. Yolk ducts not visible in all animals. Oviduct winds between ovary and junction of seminal receptacle, followed by ootype, surrounded by numerous gland cells. Uterine coils restricted between anterior extent of seminal receptacle and posterior extent of cirrus-sac. Vitellarium follicular, in two lateral parallel fields; follicles extend from posterior extremity to just anterior to ovary and level of centre of uterine coils, usually well separated from posterior border of cirrus-sac, or just reaching posterior border of cirrus-sac in few specimens, range of follicles 1652–2697 (2121); 1173–1969 (1565) from anterior extremity (pre-vitelline region), which accounts for 38.3–46.9 (42.7)% of the body length. Eggs 53–72 (62) × 30–56 (43). Excretory pore terminal. Vesicle I-shaped, anterior extent at level of ovary.

Family **Bucephalidae Poche, 1907**

Genus ***Prosorhynchus* Odhner, 1905**

Synonyms: *Chabaudtrema* Kohn, 1970, *Gotonius* Ozaki, 1924, *Paraprosorhynchus* Kohn, 1967, *Rudolphinus* Stunkard, 1974

Type species: *Prosorhynchus squamatus* Odhner, 1905

***Prosorhynchus maternus*** **Bray & Justine, 2006**

**Fig. 4:**
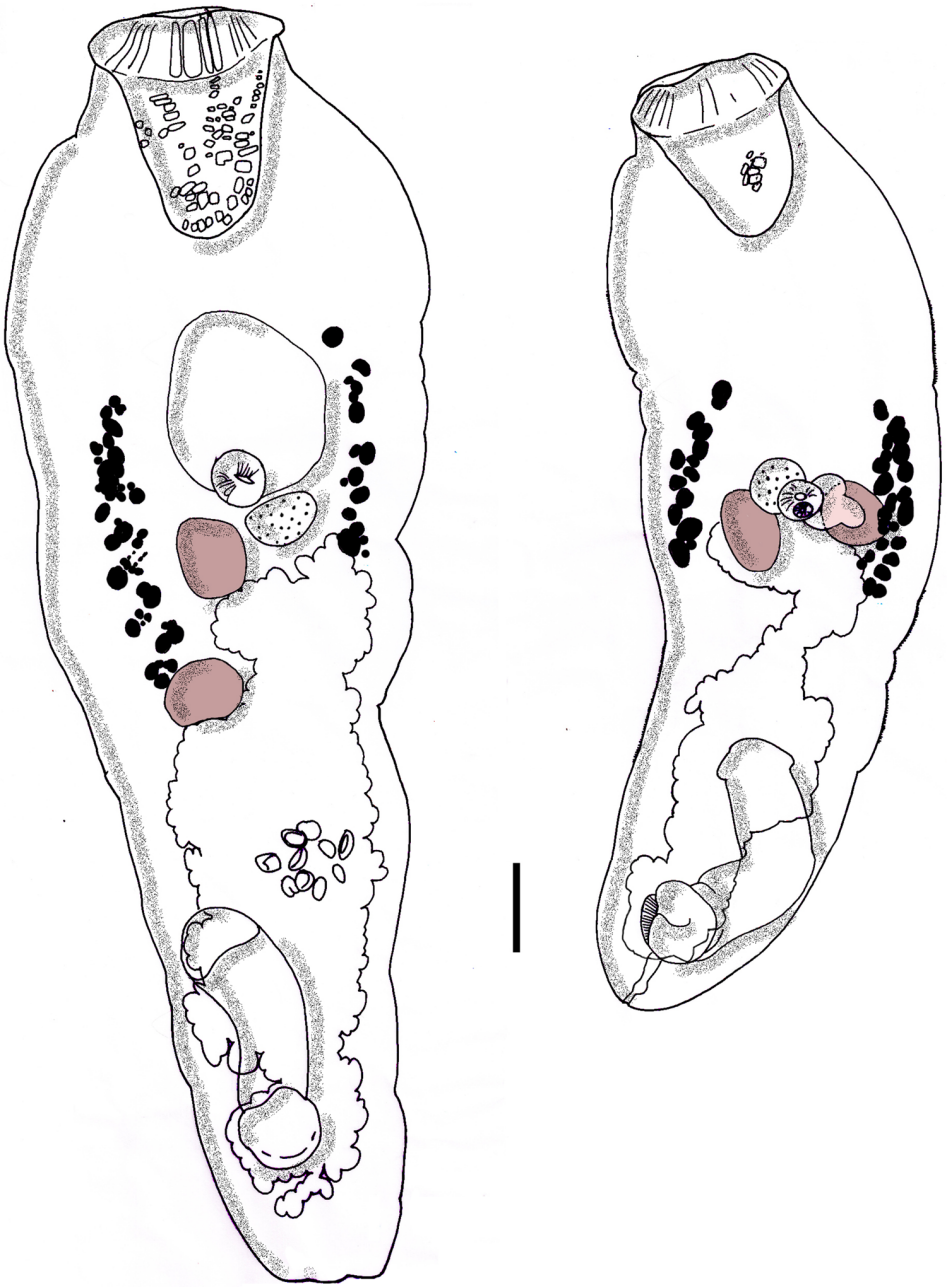
*Prosorhynchus maternus* line drawing habitus of two specimens showing intraspecific variations similar to the original species description (testes positions), uterus in outline and treated as transparent, rhynchus showing embedded gland cells, caecum either large directed anteriorly (left) or small directed laterally; scale bar: 100 µ

***Type-host:*** *Epinephelus malabaricus* (Bloch & Schneider, 1801) (malabar grouper)
***Additional host:*** * E. coioides* (Hamilton, 1822) (orange-spotted grouper)
***Type-locality:*** New Caledonia
***Additional localities:*** Vietnam, Australia, Indonesia
***Habitat:*** Intestine, pyloric caeca
***Voucher specimens and deposition:*** Zoological Museum Bogor, Indonesia, numbers MXBTr 262-265; Berlin Natural History Museum, Germany (‘Museum für Naturkunde’, catalogue ‘Entozoa’, collection ‘Vermes’)
***Infection:*** One hundred specimens (of them 40 from the intestine and 60 from the pyloric caeca) were isolated from a single fish
***Description:*** (all μm) (Fig. 4): Measurements of five gravid whole-mount worms. Body elongate, length 1103–1372 (1249), widest halfway between rhynchus and caecum, width 320–458 (375) or 29.2–33.6 (29.9)% of body length, narrows distinctly at level of posterior part of posterior testis. Tegument spinous; tiny spines reach to posterior extremity. Rhynchus slightly elongate, narrows posteriorly, muscular anterior, 177–249 (211) × 173–211 (184). Pharynx spherical, muscular, in anterior half of body. Cecum oval, sac-like, extends anteriorly from pharynx, median to vitelline fields. Testes 2, oval, in posterior part of anterior half of body, slightly separated, contiguous or slightly overlapping. Anterior testis sinistral, 50–85 (69) × 44–80 (66), slightly dextral to posterior testis. Posterior testis dextral, 41–77 (59) × 43–76 (61), in uterine-region covered by eggs. Cirrus-sac elongate, straight or curved, 252–327 (282) × 71–95 (84), muscular, in posterior part of body, never reaching posterior testis, covered by uterus. Ovary oval, 53–85 (69) × 53–79 (67), sinistral and slightly anterior to anterior testis, pre-ovarian region 436–642 (536), 43% of body length. Pre-vitelline region 340–441 (387), 31% of body length, vitelline field length 255–395 (316), 25% of body length, consists of two lateral fields of around 15 follicles, symmetrical, inside anterior half of body, not reaching the rhynchus. Uterus covers body posterior to anterior testis, pre-uterine region 478–724 (607), post-uterine region 4.1–10.1 (6.6)% of body length. Eggs numerous, tanned, 24–29 (26.8) × 12–16 (14.2). Excretory pore terminal; anterior extent of excretory vesicle not observed.

### Newly Applied Imaging Techniques for Taxonomy and Description

By using confocal laser scanning microscopy (CLSM), detailed three-dimensional (3D) images of the internal and external organs of *A. palmi* sp. nov. were obtained. Figure 2A shows the oral sucker with pharynx (dark blue), oesophagus and the intestinal bifurcation (bright green) in 3D (three axes: length, width and depth). In Fig. 2B, the connection of the pharynx, oesophagus and bifurcated intestine is clearly presented without a 3D effect, but as a cross section showing the lumen of the organs surrounded by muscle fibres. While a comparison of the thickness of the muscular regions of the pharynx and oesophagus is not possible in Fig. 2A, B shows these differentiations. The muscular ventral sucker can be seen in detail in Fig. 2C. Not only the ventral surface of the sucker is presented clearly, but also the organs more anterior to it, all in a three axes coordinate system. Similarly, the posterior part of the cirrus-sac with individual eggs surrounding it dorsally and ventrally is clearly visible in Fig. 2D. A closer look at the eggs and glandular cells anterior to ovary is given in Fig. 2E, again in 3D with all focus plains through the worm’s body. Figure 2F shows the two testes between the vitelline follicles that are positioned on various vertical levels. All these structures are presented in great detail and more cleanly than in other available microscopy techniques, further supporting the great advantages of this newly applied technique.

For a simple morphological comparison with relevant congeners, we provide the Palm patterns, (Fig. 3A, B), where the total body lengths, visualised by black bars, are detectable quickly at first glance. Similarly, the longitudinal position and extend of the organs of each species is detectable immediately (coloured bars). This is possible due to the consideration of both actual and proportional size and position of features. By adjusting the relative length of the body, the real interspecific differences along the habitus, longitudinally divided into eighths for easier comparison of organ extent, are evident. This is further simplified through the orientation with the middle of the bodies on a longitudinal axis. Therefore, these results (Fig. 3B) are an easy-to-grasp basis for the detailed interspecific comparison of relevant congeners to demarcate *Allopodocotyle palmi* sp. nov. (see “Discussion” section below).

### Phylogeny

From eight individual *Allopodocotyle palmi* sp. nov. processed, eight ITS2 sequences (302 bp length) and seven 28S sequences (1216 bp) were obtained. Sequencing of the 18S region failed.

**Fig. 5:**
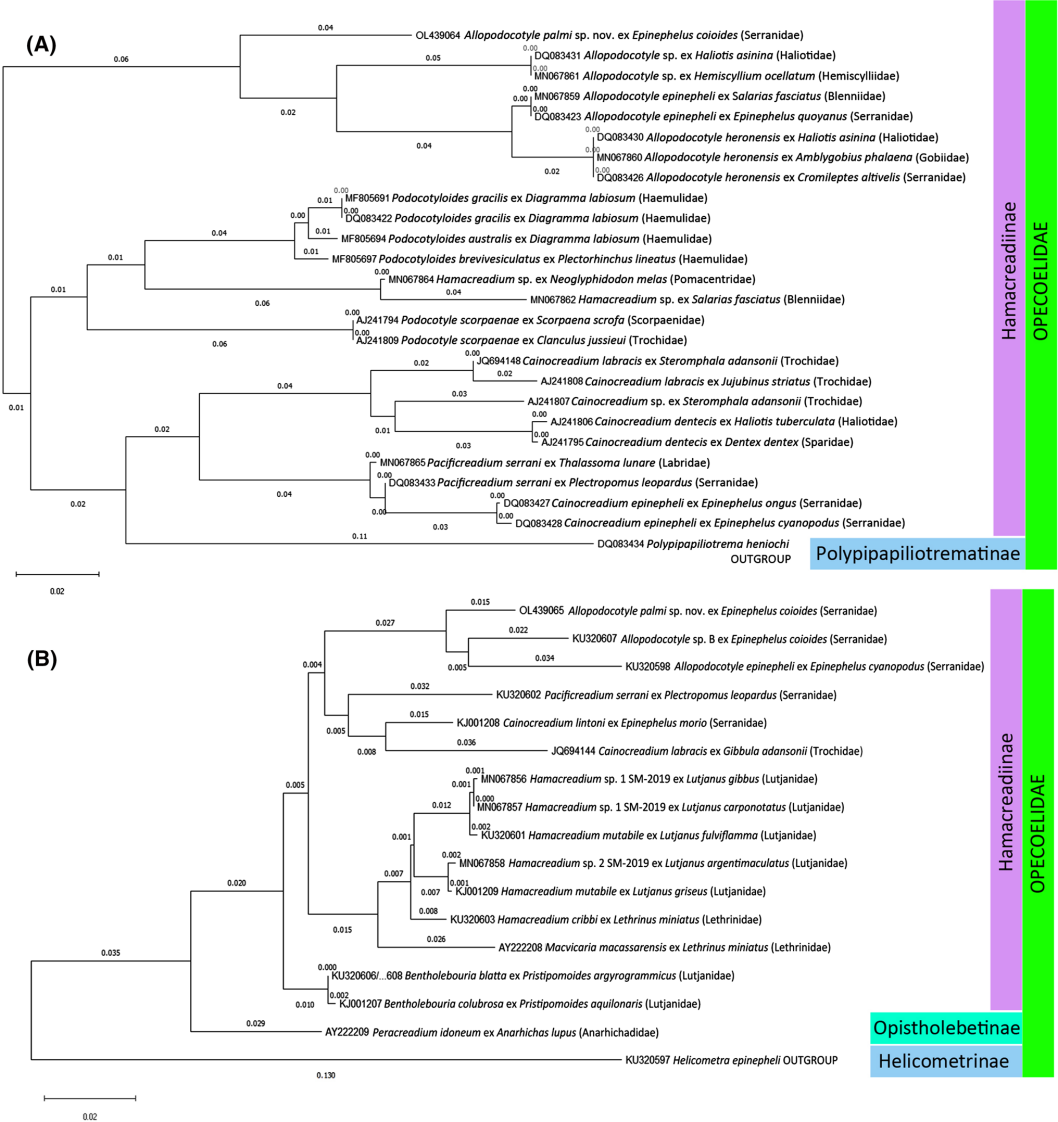
Phylogenetic trees of available *Allopodocotyle* spp. sequences and closest matches (Table 1) from NCBI BLAST. **A** ITS2 (based on the tree of [35]). **B** 28S; robustness indicated by percentage value, horizontal distances indicate substitutions per site, calculation of best fitting model and further settings see text; outgroup taxa comprised non-hamacreadiine opecoelids, specifically *Polypipapiliotrema heniochi* DQ083434 for the ITS2 analysis and *Peracreadium idoneum* AY222209 and *Helicometra epinepheli* KU320597 (originally misidentified as *H. fasciata*) for the 28S analysis

The tree for the ITS2 region includes different life-stages (Fig. 5A) and shows three major clusters. *Allopodocotyle* spp. are positioned within the first cluster on the top of the tree. Of these, the new species *Allopodocotyle palmi* stands most separate, followed by *Allopodocotyle* sp., *A. epinepheli* and *A. heronenis*. The latter two group together. The other two major clusters consist of species of *Hamacreadium*, *Podocotyle* and *Podocotyloides* on the one hand (in the centre of the figure) and of species of *Cainocreadium*, *Pacificreadium* and the non-hamacreadiine outgroup *Polypipapiliotrema heniochi* on the other hand at the bottom of the tree.

The tree for the 28S region includes different life-stages (Fig. 5B) and shows two major clusters. Species of *Allopodocotyle* group together on the top of the figure. Of these, the new species *A. palmi* stands most separate. The closest relatives to *Allopodocotyle* are species of *Cainocreadium*. The second major cluster (centre of the figure) consists of species of *Hamacreadium* and *Macvicaria macassarensis*. At the bottom of the figure, most separate standing hamacreadiines are species of *Bentholebouria*. Non-hamacreadiine opecoelid species of *Peracreadium* and *Helicometra* represent the outgroup.

The sample for *Prosorhynchus maternus* resulted in a nucleic sequence of 1031 bp length for the 28S marker (GenBank accession number OL439126). A NCBI blast identified it as this species with a percent identity (PI) of 99.2% with voucher material MH754952 from *Epinephelus coioides* from Australia, 1329 bp long. Within the coverage of 1031 bp with our material, the Indonesian worm was different to the Australian material at the six positions 189, 308, 317 (all A vs G), 508 (T vs C), 622 (G vs T), 837 (G vs A), resulting in 99.4% similarity. Further similarities were detected for *Prosorhynchus luzonicus* Velasquez, 1959 (PI: 98%), *Prosorhynchus pacificus* Manter, 1940 (97.5%), *Prosorhynchus longisaccatus* Durio & Manter, 1968 (96.2%) and to *Prosorhynchus brayi* Cutmore, Nolan & Cribb, 2018 (94.9%).

## Discussion

### Remarks on *Allopodocotyle palmi* sp. nov.

As summarised, the new species agrees well with the concept of *Allopodocotyle* Pritchard, 1966 [13, 50], i.e., *Podocotyle*-like (body elongate, unspined; ventral sucker pre-equatorial; intestinal bifurcation anterior to ventral sucker; testes post-equatorial, more or less tandem; cirrus-sac short to long, enclosing seminal vesicle; genital pore sinistral at level of oesophagus; ovary pretesticular; vitellaria normally not reaching anterior to ventral sucker; uterus pre-ovarian) but with a rounded ovary. Its distinctiveness is supported by the present molecular based phylogenetic analyses. According to detailed works, a revision and the World Register of Marine Species, *Allopodocotyle* currently comprises about 19 species and two taxa inquirenda, is characterised by a combination of character states (none of which is uniquely diagnostic), but is loosely defined and requires revision; the most convincing species are the six species known from groupers [13, 35, 48, 51, 52].

*Allopodocotyle palmi* sp. nov. is similar to the six described species of *Allopodocotyle* that infect other serranids (and a seventh not yet described species) (see above and Table 2): *A*. *epinepheli* (type-host and -locality *E. chlorostigma* Japan); *A*. *heronensis* (additional- and type-host and -locality *E. fuscoguttatus* and *Cromileptes altivelis*, Great Barrier Reef); *A*. *manteri* (type-host and -locality *E. summana*, Red Sea); *A*. *mecopera* (‘a large, spotted grouper’, Galapagos) and *A*. *plectropomi* (*Plectropomus* sp., Fiji) and *A*. *serrani* from ‘*Serranus*’ sp. from Sulawesi (as Celebes), Indonesia. According to available data [10] no species of *Serranus* Cuvier, 1816 is found in Indonesia. However, the authors assume that at the time this species was erected, *Serranus* could mean any serranid, probably a species of *Epinephelus*.

The reliability of morphological characters for differentiating opecoelid taxa has been discussed [16, 52]. The body shape might be considered useful and elongate-oval worms are common in the family Opecoelidae and especially the genus *Allopodocotyle*. Thus, a squat body as in *Allopodocotyle manteri* can be considered useful for species separation. Egg size is not useful for the separation of grouper-infecting *Allopodocotyle* spp. (see Table 2). There is the opportunity of setting the size of the vitelline follicles in relation to the egg size. The anterior extent of the vitelline follicles has been used as a generic and specific character for opecoelids, is usually fairly consistent within a species and useful for *Allopodocotyle* where the follicles reach to the ventral sucker in several species. It can help to distinguish the grouper-infecting species of the genus where it reaches only to the ovary in *A. epinepheli* and *A. plectropomi*, beyond the ovary but not to the cirrus-sac in *A. heronensis* and *A. palmi* sp. nov., and overlaps the cirrus-sac in *A. mecopera* and *A. serrani* (see Fig. 3). The restriction of the vitellarium to the hindbody separates *Allopodocotyle* from e.g., *Neolebouria* Gibson, 1976 and *Macvicaria* Gibson & Bray, 1982 [52]. In addition to the characteristics used for the key to species below, the position of the genital pore was used for species separation in congeneric descriptions.

In the following comparison with relevant congeners, we do not refer to the CLSM images, because no comparable information is available. *Allopodocotyle palmi* can be distinguished from *A*. *epinepheli* by having a larger body of 2.9–4.6 (3.7) mm vs < 2.1 mm, a cirrus-sac of 0.75–1.4 (1.1) mm vs < 0.5 mm length and testes that are separated from each other and from the ovary (Table 2 and Fig. 3). In addition, obvious differences (aside from separate vs confluent vitelline field and bifurcal vs post-bifurcal genital pore) are that *A. palmi* is more elongate and that *A. epinepheli* has a comparatively massive ventral sucker and thus *A. palmi* appears to have a more spacious forebody and greater separation between the ventral sucker and ovary. *Allopodocotyle* sp. B sensu Bray et al. [16] ex *E*. *coioides*, also from Bali, genetically different from *A*. *epinepheli* but forming a well-supported clade with it, is ‘morphologically practically indistinguishable’ and was not further described morphologically.

*Allopodocotyle epinepheli* sensu Rückert [17, 18] (from free-living and cultured Indonesian *E*. *coioides* and *E*. *fuscoguttatus*) differs from *A*. *epinepheli* sensu stricto in terms of more elongated body, sucker ratio, ratio of egg size to vitellarium vs. vitelline follicle size, separation of the vitellarium in two lateral fields, and larger distances between testes and ovary (see Table 2 and Palm patterns, Fig. 3). Downie and Cribb [13] mentioned, that her specimens resemble *A*. *heronensis* more than *A*. *epinepheli* in the elongation of the body and the separation of the gonads. However, her specimens differs from *A. heronenis* in terms of tandem vs. diagonal testes, the vitellarium (extent and separation into two lateral fields) and vitelline follicle size compared to eggs size. As shown in Fig. 3, the Palm pattern of her specimens differs from all other visualised species, including *A*. *palmi* sp. nov., because the ovary is completely restricted to the fourth eighth of the body, the anterior testis is completely restricted to the fifth eighth of the body, and the posterior testis is completely restricted to the sixth eighth of the body.

*Allopodocotyle heronensis* differs from *A*. *palmi* sp. nov. in showing tandem instead of diagonal testes (Table 2 and Fig. 3). Further, the lateral fields of the vitelline follicles are not separated and reach beyond the anterior border of the ovary. There they overlap with the posterior border of the uterus (obviously slightly separated from the ovary). The large follicles have a similar size compared to egg size. The posterior border of the cirrus-sac is clearly separated from the anterior border of the ovary. The genital pore is at the level of the intestinal bifurcation. *Allopodocotyle manteri* differs greatly from its grouper-infecting congeners. It has a squat, pyriform body and the organs are differently positioned, e.g., this is the only of the here mentioned species that has the uterine coils and the vitelline follicles entirely in the second body half. The cirrus-sac and ventral sucker reach the second half of the body only in this species. Similarly, all other organs are positioned more posterior in this species, e.g., the ovary and testes in the last third of the body. The published body sizes in the original species description text (≤ 1.75 mm) are smaller than as calculated from the associated figure and scale bar (> 2 mm). For its Palm pattern we refer to its original line drawing and its scale.

*Allopodocotyle mecopera* differs from *A*. *palmi* sp. nov. especially in showing tandem instead of diagonal testes (Table 2 and Fig. 3). Further, the two lateral fields of the vitellary follicles of this species are confluent (at least dorsally), give the appearance of not being divided (except at the level of the ovary) and reach just to the anterior border of the ovary. There they overlap with the posterior border of the uterus (obviously not separated from the ovary); and the follicles have about 60–80% of the egg size. With a minimum length of 70 µm, eggs of *A. mecopera* are the largest amongst the grouper-infecting congeners, who show maximum egg sizes of 70 µm. The posterior border of the cirrus-sac is also at the level of the anterior border of the ovary. The genital pore lies at the level of the intestinal bifurcation.

*Allopodocotyle plectropomi* has the vitellarium well separated into two lateral fields and reaching just to the level of the centre of the ovary (but not to its anterior border nor farther). The posterior border of the uterus is not separated from the ovary but at the level of its anterior border; and the follicles are about 50% of the egg size. The posterior border of the cirrus-sac is well separated from the anterior border of the ovary. The genital pore lays well anterior to the level of the intestinal bifurcation right at the level of the junction of the pharynx and oesophagus. This species differs from *A*. *palmi* sp. nov. in having the ovary and anterior testis in the first half of body (vs ovary on border from first to second half of body and testis in second half of body), uterine coils restricted to the third eighth of body (vs fourth) (Table 2 and Fig. 3).

*Allopodocotyle serrani* has the vitellarium well divided into two lateral fields and reaching from close to the posterior extremity to the centre of the broad part of the cirrus-sac, about midway between the ventral sucker and the ovary; and the follicles have about 50% of the egg size. The posterior border of the uterus is well separated from the ovary and lies halfway between the anterior border of the ovary and the posterior border of the cirrus-sac. The genital pore is well posterior to the level of the intestinal bifurcation. This species differs from *A*. *palmi* sp. nov. in having a longer cirrus-sac, covering one third of body length (1.5–1.9 vs 0.75–1.4 (1.1) mm), distinctly overlapping the anterior region of vitelline fields, and almost reaching as far as posterior border of uterine coils. Thus, the uterine coils and vitellarium only overlap the cirrus-sac in *A. palmi*. In *A. serrani*, the ovary is on the border of first and second halves of body, while in *A. palmi* it is entirely in the second half of body (Table 2 and Fig. 3). Other organ length and positions are similar to those of *A*. *palmi* sp. nov., however, despite similar body length, *A*. *serrani* is much wider.

### Key to Species of *Allopodocotyle* Infecting Serranid Groupers

Detailed keys to the *Allopodocotyle* species are available [53]. They have been split up in the morphological groups A, B and C, mainly based on testes positions and cirrus-sac length. However, the six serranid-infecting species are divided into group A (testes diagonal, cirrus-sac long, extending from well anterior of ventral sucker to well posterior to it; *A*. *epinepheli*, *A*. *manteri*, *A*. *plectropomi* and *A*. *serrani*) and group C (tandem testes; *A*. *heronensis* and *A*. *mecopera*). None is in group B (testes diagonal, cirrus-sac just overlapping the anterior margin of the ventral sucker). The most convincing species of *Allopodocotyle* are from groupers, therefore the system of three morphologically different groups does not reflect phylogeny. The provided key therefore just includes these phylogenetically most related species.

**Table:** 

1	(A) Testes tandem	2
	(B) Testes diagonal	3
2	(A) Body 1.9–3.3 × 0.2–0.4 mm elongate; sucker ratio 1:1.9; cirrus-sac < 0.5 mm; testes very well separated (also from ovary), almost as wide as body	*A. heronensis*
	(B) Body 1.9–2.5 × 0.5–0.6 mm; sucker ratio 1: > 2; cirrus-sac 0.75 mm; testes just slightly separated (also from ovary), each half as wide as body	*A. mecopera*
3	(A) Body squat; uterus in posterior half of body; ovary and testes in posterior third of body	*A. manteri*
	(B) Body elongate; uterus in anterior half of body; ovary and testes in middle third of body	4
4	(A) Body < 2.1 mm; sucker ratio 1:2.4; cirrus-sac < 0.5 mm; testes almost contiguous (also almost contiguous to ovary), each less than 1/3 wide as body	*A. epinepheli*
	(B) Body > 2.7 (2.4 for an outlier) mm; sucker ratio 1: < 2.4; cirrus-sac > 0.5 mm	5
5	(A) Ovary and anterior testis in first half of body; uterine coils restricted to third eighth of body (body 2.7 (2.4 for an outlier)–3.5 mm long; sucker ratio 1:1.5–1.7; cirrus-sac 0.9 mm; testes very well separated (also from ovary), each 1/5 wide as body)	*A. plectropomi*
	(B) Ovary mainly and anterior testis completely in posterior half of body; uterine coils in fourth eighth of body	6
6	(A) Cirrus-sac 1.5–1.9 mm, posteriorly distinctly overlapping with anterior region of vitellarium, and almost reaching as far as posterior boarder of uterine coils (= uterine coils and vitellarium overlapping cirrus-sac); ovary in posterior half of body,	*A. serrani*
	(B) Cirrus-sac 0.75–1.4 mm, posteriorly well separated from anterior region of vitellarium, not reaching anterior boarder of uterine coils (= coils and vitellarium not overlapping cirrus-sac); ovary in about mid-body	*A. palmi* sp. nov.

### Remarks on *Prosorhynchus maternus*

Based on the phylogenetic analyses, the closest relatives to *P. maternus* are *P. luzonicus* and *P. pacificus*. Both species differ from *P. maternus* morphologically as described by Bray and Justine [54].

The average length of our specimens is almost double those of the original description from *E. malabaricus*, but is similar to the findings of voucher material of this species from Australian *E. coioides* [54, 55]. The morphometry and molecular data (GenBank accession number OL439126, 28S sequence) agree with the reference material for the species [54, 55]. We consider the 0.6% sequence differences in the 28S marker as uninformative single point mutations and, likewise, the slight morphological differences as intraspecific. The species is newly recorded for Indonesia, and the third record for *Epinephelus coioides*, from which it was isolated in Vietnamese and Australian waters [55, 56].

### Advantages of the Newly Introduced Imaging Methods

An integrated approach, namely applying light microscopy with photography and drawing, the Palm pattern system, but also confocal laser scanning microscopy as well as phylogenetic analyses of various gene markers, is herein considered as useful for digenean species descriptions. Especially the imaging by the confocal laser scanning is a valuable tool and can be used for future taxonomic Digenea descriptions.

The CLSM allows a detailed view of the internal and external organs or of the surface of whole worms. Theisen et al. [57, 58] applied this methodology to parasitic helminths. It improved characterisation of the position of structures, providing different angles of view for illustrating and comparison as well as accurate measurements along 3 axes (length, width and depth). This methodology was suitable to detect minor, often difficult to describe differences in structures of such small fish parasitic helminths. In contrast to LM, the composite image stacks allow multiple plains to be seen in focus, providing a better understanding of the location and connection of the individual organs. The description of the digenean *Didymocystis lamotheargumedoi* Kohn and Justo, 2008 contains black/white CLSM photos (their Fig. 1D–F) [59]. The manuscript refers to Fig. F only, with half a sentence. As Neitemeier-Duventester et al. [30] suspected, the examination of Digenea is not only possible with the CLSM, but has some advantages. It can be directly applied on preparations originally prepared for the LM, e.g., carmine stained and Canada balsam mounted worms. There is no additional effort and even decade-old museum material (cestodes) have been successfully described with CLSM. Museum collections of Digenea are usually similarly stained and mounted. This offers the chance to further analyse them accordingly.

A third dimension is without doubt of advantage and the possibility to analyse (i) surface and (ii) inner organs of just one single mounted worm under one single microscope (instead of preparing a worm for LM, another one for SEM, and probably—even though applied rarely in taxonomy—a third one for transmission electron microscopy (TEM)) is of great advantage.

For our analyses, no special filter was applied and the colour-based differentiation of the organ is a result of the carmine staining and the applied laser wavelengths. Here, chromatically opposite filters that provide anaglyph 3D images with a stereoscopic effect supported by color-coded anaglyph glasses (red/cyan shift) were not applied. Software solutions can easily provide such red/cyan shifts that are meant to be viewed with such glasses. Movies with rotating 3D models are not provided because the described platyhelminth is especially flat and not equipped with exposed armature except the ventral sucker that would justify them. Our species description, with an introduction to the newly applied method, is therefore based on providing CLSM images for comparison with commonly presented DIC light micrographs to demonstrate the direct benefits of CLSM. As visible in our figures, in contrast to the limited LM, the 3D images allow to show the morphological characteristics in a three axes coordinate system. This allows measurement of length, width and depth of one single dorsoventrally (or in any other orientation) mounted worm in all directions simultaneously. All focus levels of the organs are provided in a sharp figure, whereas LM can only provide an image of one focus plane of a mounted worm. Software solutions for LM can also produce multiple layer figures achieved by focussing vertically through the whole mount worm while recording, but the calculated figure results in invisible overlaid structures and the display of optical noise and artefacts.

The newly erected Palm pattern system can support and simplify a morphological comparison of related taxa. The Palm pattern is suitable for detailed comparison of morphologically similar Digenea taxa and other organs can be considered, depending on the morphology of the investigated families (e.g., glandular and aglandular extension of the pars prostatica). Further, it might be considered to construct not only relative and absolute Palm patterns for the longitudinal length and organ positioning, but also transverse (body width and transverse position of organs, e.g., presentation of a sinistral or dextral ovary or genital pore or diagonal testes). In using the Palm pattern several caveats should be borne in mind. The possible effects of natural variation, variation due to different methods of fixation (particularly flattening), different conditions of collection (fresh, frozen, etc.) and allometric growth [cf. 60, 61] should be considered. However, all this has to be considered for other imaging techniques as well. Nevertheless, as the number of similar species within genera increases a clear graphical system such as the Palm pattern will facilitate easier identification.

### Phylogenetic analyses of *Allopodocotyle palmi* sp. nov.

The phylogenetic trees (Fig. 5A, B) show that *A. palmi* sp. nov. is a distinct species, belonging to the hamacreadiine opecoelids. Both the ITS2 and 28S trees show that *A. palmi* is basal relative to all other sequenced *Allopodocotyle*. The genus parasitises groupers as finals hosts and have, similar to all Hamacreadiinae, fishes as second intermediate hosts [35]. Haliotidae and Trochidae function as first intermediate host, and second host fishes of the species which infect groupers are e.g., blennies and gobies (Fig. 5A, B and [35]). It is known that most cercariae of opecoelids, including those known for the Hamacreadiinae are cotylocercous, meaning they cannot swim but crawl so that small fishes on or near the benthos or among coral are probably indiscriminately exploited [35].

*Podocotyle* and *Podocotyloides* (ITS) as well as *Cainocreadium* and *Pacificreadium* (28S) are the closest relatives of *Allopodocotyle*. A species referred to *Allopodocotyle* according its GenBank entry (Fig. 5A, DQ083422) has never been described as such, and represents *Podocotyloides gracilis* based on our analyses.

## Conclusion

Although several articles on marine Indonesian fish parasites have been published within the last decades, the current knowledge is still poor, with an estimation of 5.5% of the local marine teleost fish parasite fauna being known [2]. We herewith enhance the biodiversity knowledge from Indonesian waters by a newly described marine parasite species and a new locality record of a known species. The grouper *E. coioides* is the best investigated fish species (from both free-living natural stocks as well as mariculture farms) in Indonesia concerning parasites, resulting from its high value for human consumption [2]. It was therefore already pointed out as a local model organism for fish parasitology that shows specific characteristics and advantages that make it of interest for scientific questions and allow simple access to investigate certain individual aspects, and compare them with available information. This study demonstrates that even the fish species with the highest number of individuals investigated can still bear a new parasite species and additional locality record in this marine biodiversity hotspot.

## Supplementary Information

Below is the link to the electronic supplementary material.

Supplementary file1 Additional file 1: Table S1. Morphometry measures and raw data of 22 reference individuals (XLSX 18 KB)

## Data Availability

Datasets are either deposited in publicly available repositories or presented in the main manuscript or additional supporting files.
